# Supplementary material for: Highly sensitive scent-detection of COVID-19 patients in vivo by trained dogs
Source: PLoS One. 2021 Sep 29;16(9):e0257474. doi: 10.1371/journal.pone.0257474 (PMC8480816; doi:10.1371/journal.pone.0257474)
Supplement: S1 File — (DOCX) [file pone.0257474.s001.docx]

S1 File

**Highly Sensitive Scent-Detection of COVID-19 Patients by Trained Dogs**

Omar Vesga^1,2*^, Maria Agudelo^1,2^, Andrés F. Valencia-Jaramillo^2,3^, Alejandro Mira-Montoya^2,3^, Felipe Ossa-Ospina^2,3,4^, Esteban Ocampo^3^, Karl Čiuoderis^5^, Laura Pérez^5^, Andrés Cardona^5^, Yudy Aguilar^2^, Yuli Agudelo^1^, Juan P. Hernández-Ortiz^5,6^, Jorge E. Osorio^5,6^.

*Corresponding author: Omar Vesga, MD. [omar.vesga@udea.edu.co](mailto:omar.vesga@udea.edu.co)

Professor Omar Vesga, MD. Head and Attending Physician, Section of Infectious Diseases, Hospital Universitario San Vicente Fundación. Director GRIPE and Professor of Medicine, University of Antioquia Medical School.

Maria Agudelo, MD, PhD. Attending Physician, Section of Infectious Diseases, Hospital Universitario San Vicente Fundación. Scientific Director, GRIPE, University of Antioquia.

Andrés F. Valencia-Jaramillo, DVM. Director of Training, Colina K-9. Assistant Researcher GRIPE.

Alejandro Mira-Montoya, DVM. Trainer, Colina K-9. Assistant Researcher GRIPE.

Felipe Ossa-Ospina. Trainer, Colina K-9. Undergraduate student , School of Veterinary Medicine, and research-student at GRIPE, University of Antioquia.

Esteban Ocampo. Trainer, Colina K-9.

Karl Čiuoderis, DVM - PhD (cand.). Associate Scientist, Colombia/Wisconsin One-Health Consortium, Universidad Nacional de Colombia, Sede Medellín

Laura Pérez, M.Sc. Biology. Associate Scientist, Colombia/Wisconsin One-Health Consortium, Universidad Nacional de Colombia, Sede Medellín

Andrés Cardona, M.Sc. Biology. Associate Scientist, Colombia/Wisconsin One-Health Consortium, Universidad Nacional de Colombia, Sede Medellín

Yudy Aguilar, PhD (cand.). Associate Scientist, GRIPE, University of Antioquia.

Yuli Agudelo, MD. Director of Clinical Management, Hospital Universitario San Vicente Fundación, sede Medellín.

Professor Juan P. Hernández-Ortiz, PhD. Director Colombia/Wisconsin One-Health Consortium, Universidad Nacional de Colombia, Sede Medellín.

Professor Jorge E. Osorio, DVM, PhD. Director Colombia/Wisconsin One-Health Consortium, University of Wisconsin-Madison.

^1^Section of Infectious Diseases, Hospital Universitario San Vicente Fundación, Medellín, Colombia.

^2^GRIPE, Universidad de Antioquia, Medellín, Colombia.

^3^Colina K-9, La Ceja, Colombia.

^4^Undergraduate School of Veterinary Medicine, Universidad de Antioquia, Medellín, Colombia.

^5^Colombia/Wisconsin One-Health Consortium, Departamento de Materiales, Facultad de Minas, Universidad Nacional de Colombia, Sede Medellín, Colombia.

^6^Department of Pathobiology, School of Veterinary Medicine, University of Wisconsin, Madison, USA.

Table of Contents

[Supplementary Figures 4](#_Toc80945411)

[S1 Fig. Experimental set up to determine biosafety of SARS-CoV-2 containment devices. 4](#_Toc80945412)

[S2 Fig. Phase 2: in vitro diagnosis. 5](#_Toc80945413)

[Supplementary videos 6](#_Toc80945414)

[S1 Video. Canine scent-detection: in vitro diagnosis. 6](#_Toc80945415)

[S2 Video. Canine scent-detection: in vivo screening under normal-life conditions (effectiveness trial). 7](#_Toc80945416)

[Supplementary Tables 8](#_Toc80945417)

[S1 Table. Sample size and experimental design. 8](#_Toc80945418)

[S2 Table. Phase 1: in vitro recognition results. 9](#_Toc80945419)

[S3 Table. Phase 2: in vitro diagnosis results. 10](#_Toc80945420)

[S4 Table. Phase 3: in vivo screening (efficacy trial). 11](#_Toc80945421)

[S5 Table. Phase 4: effectiveness assay to determine dog performance during in vivo screening under real-life conditions. 12](#_Toc80945422)

[S6 Table. In vitro determination of the limit of detection of SARS-CoV-2 by 4 canines. 13](#_Toc80945423)

[S7 Table. Biosafety data for dogs, trainers, and physicians involved in sampling, experimentation, and medical care of COVID-19 patients. 14](#_Toc80945424)

[S8 Table. Biosafety data. Testing the devices used to contain SARS-CoV-2 specimens. 15](#_Toc80945425)

# Supplementary Figures

**S1 Fig. Experimental set up to determine biosafety of SARS-CoV-2 containment devices.** Each cage had 3 immunocompetent Syrian hamsters (*Mesocrisetus auratus*) exposed during 4 days to SARS-CoV-2 enclosed in device one (D1; Experimental Group 1 and Control Group A) or two (D2; Experimental Group 2 and Control Group B). The fifth cage had a sterile gauze impregnated with SARS-CoV-2 (Control Group B). Hamsters in Experimental Groups could sniff the virus inside D1 and D2, but a mesh cage prevented them from touching or interacting with the devices, while those in Control Groups could sniff, touch, play, lick, bite, or eat D1, D2, and the virus-impregnated gauze.


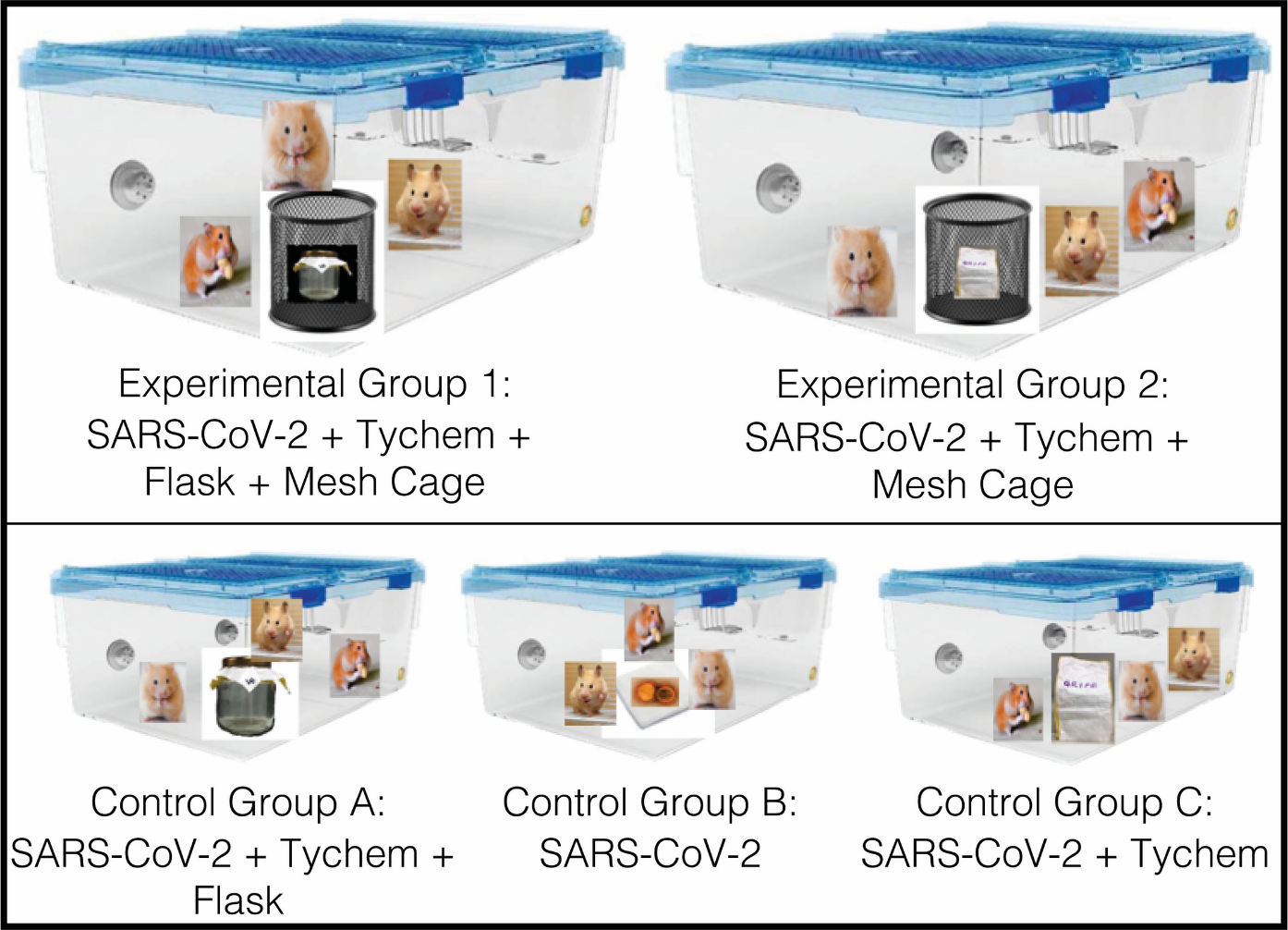


**S2 Fig. Phase 2: in vitro diagnosis.** Quantification of canine performance detecting COVID-19 in vitro by scenting saliva samples; n 6000, prevalence 2.2%. Empty symbols represent the different dogs, while the black circle is for all 6 dogs. The vertical lines above and below the symbols represent the 95% confidence interval, which is contained within the symbol for *SPC* and *NPV*.

**
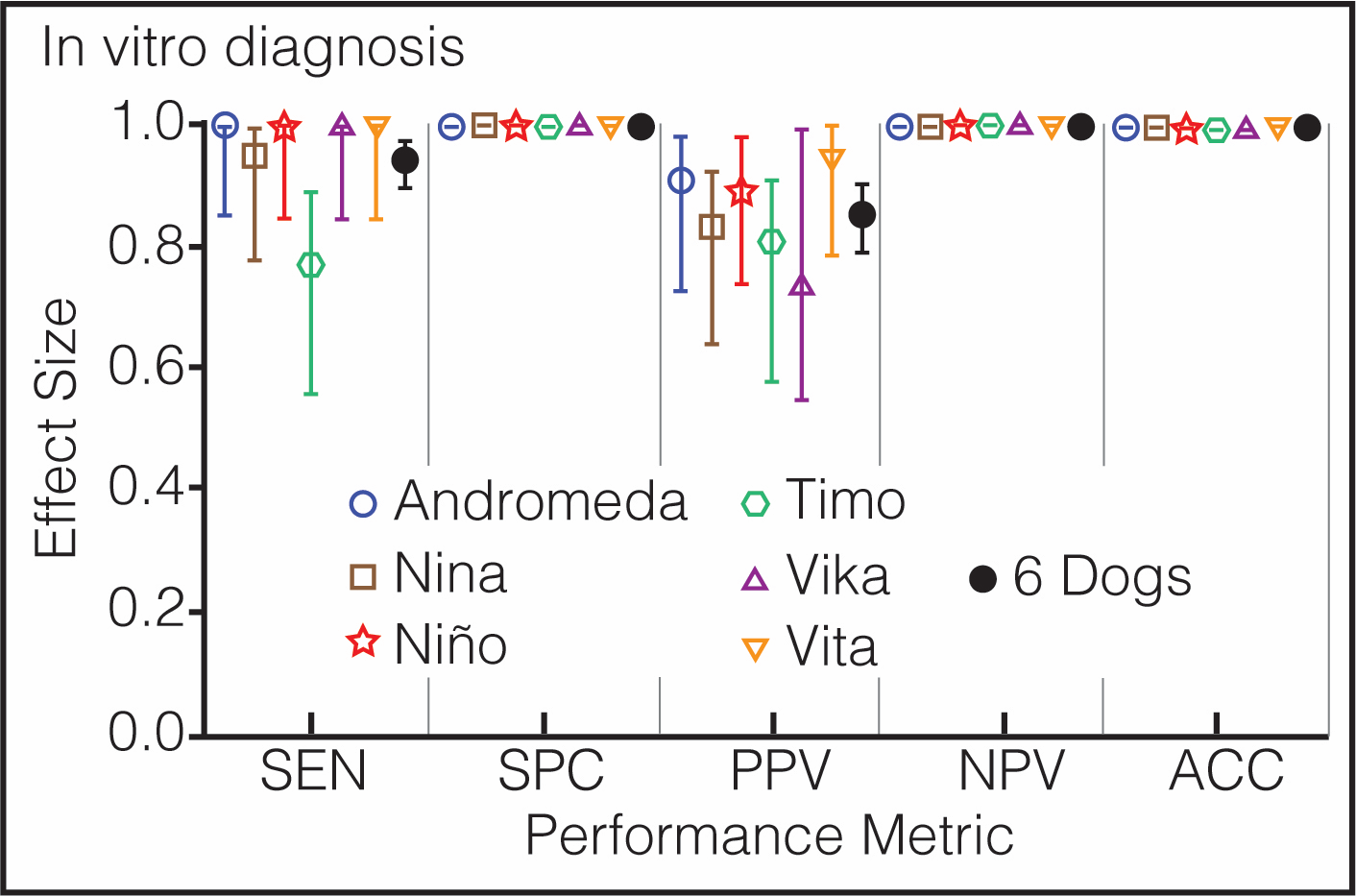
**

# Supplementary videos

All relevant data are in the paper and its Supporting Information files. Relevant video files have been uploaded to Figshare and are available at:

<https://doi.org/10.6084/m9.figshare.14815848.v1>

## **S1 Video. Canine scent-detection: in vitro diagnosis.**

**
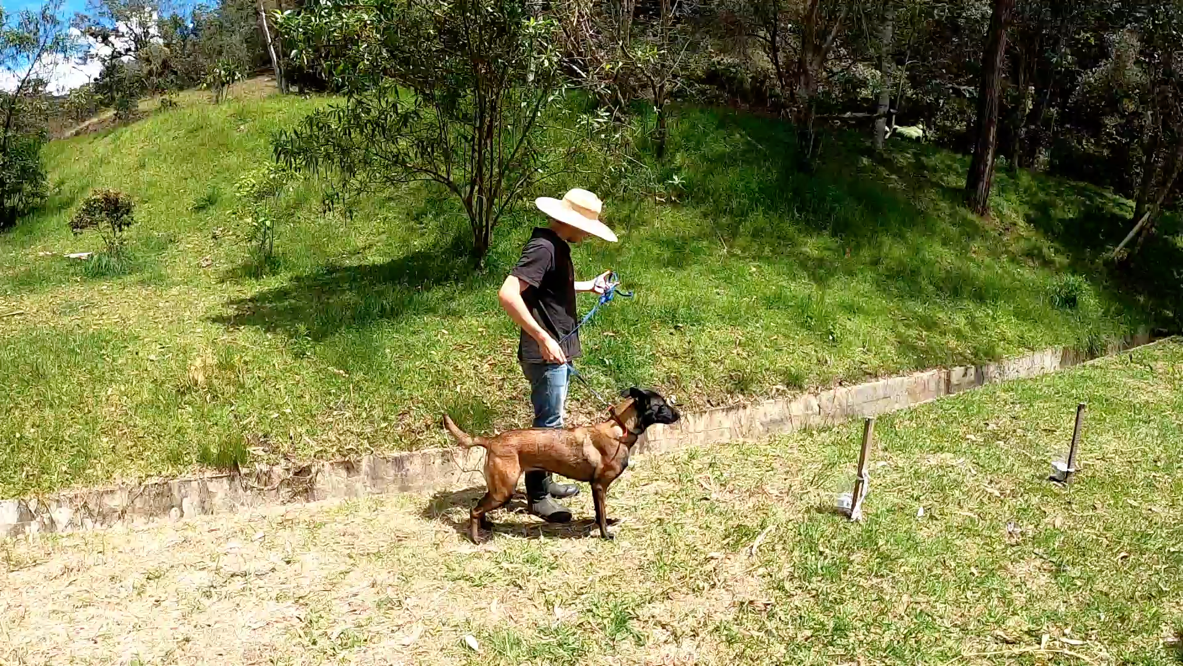
**

This video, edited to hide human faces, illustrates the experimental field as described in the text, and the dog performance during a phase 2 trial. The trainer is not aware of the position or the prevalence of the samples with positive stimulus, but the director of training does. The set up in the video was the same for all in vitro experiments, an open field with 100 flasks containing saliva from COVID-19 Patients 4-12 or, in phase 2, saliva from healthy ambulatory citizens (Patients 13-112). Vika is displaying a perfect performance during one of the experiments for *in vitro* diagnosis, in which prevalence was set randomly at 4%. To watch the video, click on this link: <https://doi.org/10.6084/m9.figshare.14815848.v1>

## **S2 Video. Canine scent-detection: in vivo screening under normal-life conditions (effectiveness trial).**

**
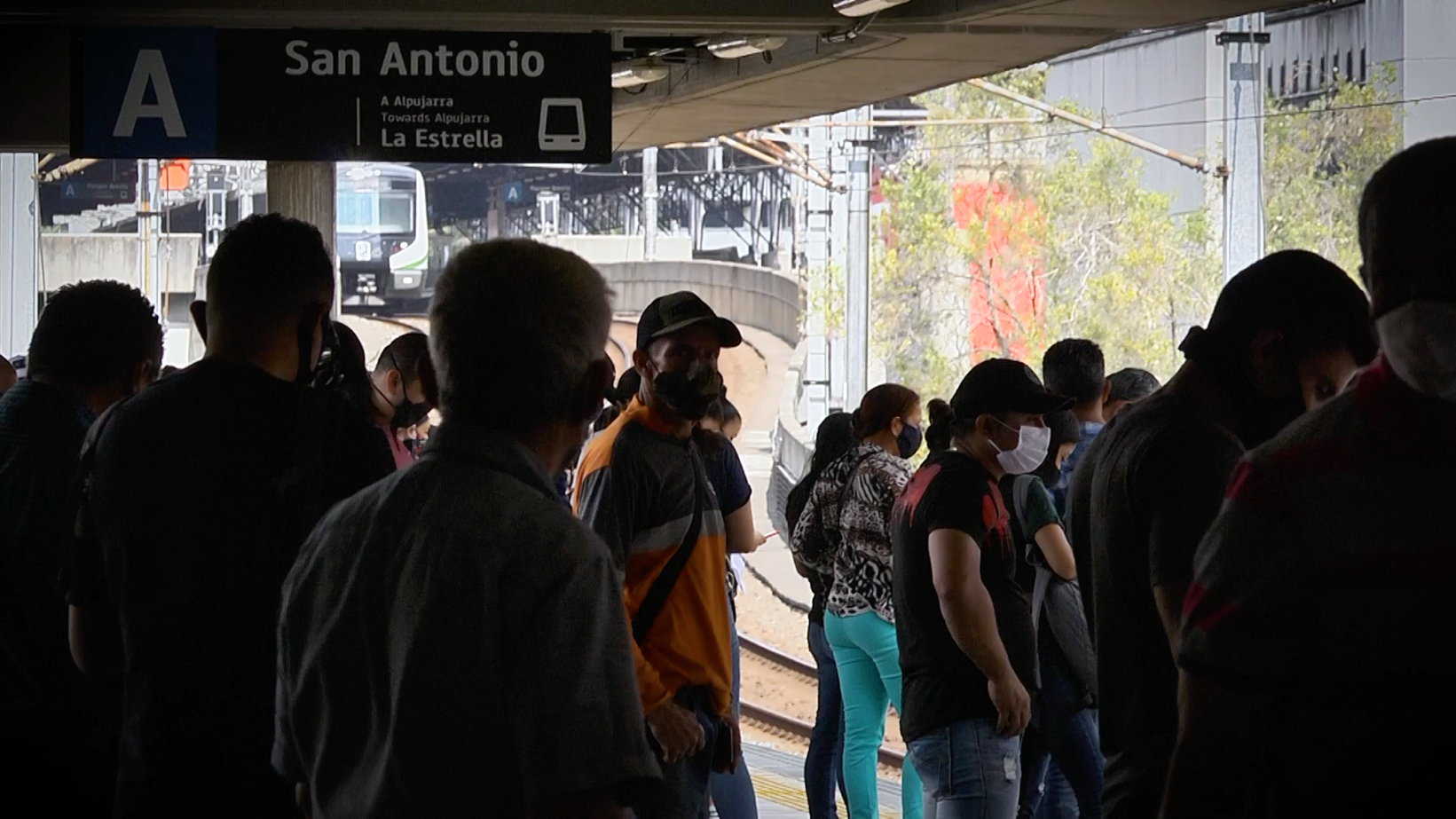
**

The intention of this video is to show the real-life scenario where the effectiveness trial was executed. The Medellin’s Metro System transports 1.5 million passengers every day, and the station in the video (San Antonio) has, on average, 60,000 users at any given time during business hours. The COVID-19 status of the participants was unknown to everybody, trainers were not aware of the experiment date or location, and dogs had no special place to work, nor received additional training or habituation to this particular environment before the experiments, which included 550 consecutive patients over two days. People of all ages requested enthusiastically to participate in the trial, as dogs play a prominent role in Colombian society and life-style. To watch the video, click on this link: <https://doi.org/10.6084/m9.figshare.14815848.v1>

# Supplementary Tables

**S1 Table. Sample size and experimental design.** Prevalence frequencies expected when the study was designed and those obtained from the data, with the respective sample size calculations for each experimental phase.

| **Experimental Phase** | **Expected Prevalence** | **Significance Level** | **Power** | **Null Hypothesis** | **Alternative Hypothesis** | **n required for SENSITIVITY** | **n required for SPECIFICITY** | **Study Population** | **COVID-19 Prevalence** |
| --- | --- | --- | --- | --- | --- | --- | --- | --- | --- |
| In vitro recognition | 0.05 | 0.05 | 0.8 | 0.8 | 0.9 | 2140 | 113 | 3200 | 0.0756 |
| In vitro diagnosis | 0.05 | 0.05 | 0.8 | 0.8 | 0.9 | 2140 | 113 | 6000 | 0.0220 |
| In vivo screening, low-risk group | 0.05 | 0.05 | 0.8 | 0.6 | 0.9 | 380 | 20 | 320 | 0.0125 |
| In vivo screening, medium-risk group | 0.10 | 0.05 | 0.8 | 0.6 | 0.9 | 190 | 21 | 259 | 0.0270 |
| In vivo screening, high-risk group | 0.30 | 0.05 | 0.8 | 0.6 | 0.9 | 63 | 27 | 269 | 0.3010 |
| In vivo screening, all subjects | 0.10 | 0.05 | 0.8 | 0.7 | 0.9 | 310 | 34 | 848 | 0.1080 |

**S2 Table. Phase 1: in vitro recognition results.** Performance metrics of six canines after four weeks of scent-detection training. The dogs sniffed 100 flasks/experiment containing sterile 0.9% saline solution or SARS-CoV-2. The position and prevalence of the virus was randomized for each dog. The trainers knew both variables for most experiments.

| **Metric** | **Dog Name (breed*), Effect Size of each Performance Metric [95% Confidence Intervals], and P value (Latent Class Analysis)** | | | | | | |
| --- | --- | --- | --- | --- | --- | --- | --- |
|  | **Andromeda (BM)** | **Nina (BM)** | **Niño (PB)** | **Timo (BM)** | **Vika (BM)** | **Vita (AMxSH)** | **All 6 Dogs** |
| **Prevalence (%)** | 5.00 | 8.60 | 7.00 | 6.50 | 5.57 | 8.50 | 7.56 |
| **n** | 100 | 1000 | 200 | 200 | 700 | 1000 | 3200 |
| **TP** | 5 | 75 | 13 | 11 | 36 | 75 | 215 |
| **TN** | 93 | 893 | 181 | 185 | 653 | 877 | 2882 |
| **FP** | 2 | 21 | 5 | 2 | 8 | 38 | 76 |
| **FN** | 0 | 11 | 1 | 2 | 3 | 10 | 27 |
| ***SEN* (%) [95% C.I.]** | 100 [56.6-100] | 87.2 [78.5-92.7] | 92.9 [68.5-99.6] | 84.6 [57.8-97.3] | 92.3 [79.7-97.4] | 88.2 [79.7-93.5] | 88.8 [84.3-92.2] |
| ***SPC* (%) [95% C.I.]** | 97.9 [92.7-99.7] | 97.7 [96.5-98.5] | 97.3 [93.9-98.9] | 98.9 [96.2-99.8] | 98.8 [97.6-99.4] | 95.8 [94.4-97.0] | 97.4 [96.8-97.9] |
| ***PPV* (%) [95% C.I.]** | 71.4 [35.9-94.9] | 78.1 [68.9-85.2] | 72.2 [49.1-87.5] | 84.6 [57.8-97.3] | 81.8 [68.0-90.5] | 66.4 [57.3-74.4] | 73.9 [68.6-78.6] |
| ***NPV* (%) [95% C.I.]** | 100 [96.0-100] | 98.8 [97.8-99.3] | 99.5 [97.0-100] | 98.9 [96.2-99.8] | 99.5 [98.7-99.9] | 98.9 [97.9-99.4] | 99.1 [98.7-99.4] |
| ***ACC (%)*** | 98.0 | 96.8 | 97.0 | 98.0 | 98.4 | 95.2 | 96.8 |
| ***LR*** | 47.5 | 38.0 | 34.5 | 79.1 | 76.3 | 21.3 | 34.6 |
| ***P*** | <0.0001 | <0.0001 | <0.0001 | <0.0001 | <0.0001 | <0.0001 | <0.0001 |

*BM: Belgian malinois; PB: Pit bull; AMxSH: Alaskan malamute by Siberian husky first generation cross.

**S3 Table. Phase 2: in vitro diagnosis results.** Performance metrics of six canines after seven weeks of scent-detection training. The dogs sniffed 100 flasks/experiment containing saliva from healthy human subjects or SARS-CoV-2 positive patients. The position of the virus was randomized for each dog, and prevalence was fixed at 2.2%. The trainers were blinded about the position of the positive samples in the field for all experiments.

| **Metric** | **Dog Name (breed*), Effect Size of each Performance Metrics [95% Confidence Intervals], and *P* value (Latent Class Analysis)** | | | | | | |
| --- | --- | --- | --- | --- | --- | --- | --- |
|  | **Andromeda (BM)** | **Nina (BM)** | **Niño (PB)** | **Timo (BM)** | **Vika (BM)** | **Vita (AMxSH)** | **All 6 Dogs** |
| **Prevalence (%)** | 2.20 | 2.20 | 2.20 | 2.20 | 2.20 | 2.20 | 2.20 |
| **n** | 1000 | 1000 | 1000 | 1000 | 1000 | 1000 | 6000 |
| **TP** | 22 | 21 | 22 | 17 | 22 | 22 | 126 |
| **TN** | 976 | 974 | 976 | 974 | 970 | 977 | 5847 |
| **FP** | 2 | 4 | 2 | 4 | 8 | 1 | 21 |
| **FN** | 0 | 1 | 0 | 5 | 0 | 0 | 6 |
| ***SEN* (%) [95% C.I.]** | 100 [85.1-100] | 95.5 [78.2-100] | 100 [85.1-100] | 77.3 [56.6-89.9] | 100 [85.1-100] | 100 [85.1-100] | 95.5 [90.4-97.9] |
| ***SPC* (%) [95% C.I.]** | 99.8 [99.3-100] | 99.6 [99.0-99.8] | 99.8 [99.3-100] | 99.6 [99.0-99.8] | 99.2 [98.4-99.6] | 99.9 [99.4-100] | 99.6 [99.5-99.8] |
| ***PPV* (%) [95% C.I.]** | 91.7 [74.2-98.5] | 84 [65.4-93.6] | 91.7 [74.2-98.5] | 81 [60.0-92.3] | 73.3 [55.6-85.8] | 95.7 [79.0-99.8] | 85.7 [79.2-90.5] |
| ***NPV* (%) [95% C.I.]** | 100 [99.6-100] | 99.9 [99.4-100] | 100 [99.6-100] | 99.5 [98.8-99.8] | 100 [99.6-100] | 100 [99.6-100] | 99.9 [99.8-100] |
| ***ACC (%)*** | 99.8 | 99.5 | 99.8 | 99.1 | 99.2 | 99.6 | 99.6 |
| ***LR*** | 489.0 | 233.4 | 489.0 | 188.9 | 122.3 | 978.0 | 266.7 |
| ***P*** | <0.0001 | <0.0001 | <0.0001 | <0.0001 | <0.0001 | <0.0001 | <0.0001 |

*BM: Belgian malinois; PB: Pit bull; AMxSH: Alaskan malamute by Siberian husky first generation cross.

**S4 Table. Phase 3: in vivo screening (efficacy trial).** Performance metrics of five canines after 13 weeks of scent-detection training. Dogs scent-interrogated 848 subjects that included 269 hospitalized patients, 259 health-care workers from the same institution, and 320 government employees; n varies slightly between dogs because a participant did not show up for the dog test or the dog refused to interrogate an individual. Dog trainers were blinded regarding the COVID-19 status of all subjects. P values (Fisher’s Exact Test) express the probability that the dogs identified the patients infected by SARS-CoV-2 by chance alone.

| **Metric** | **Dog Name (breed*) and Effect Size of each Performance Metric [95% Confidence Intervals]** | | | | | |
| --- | --- | --- | --- | --- | --- | --- |
|  | **Andromeda (BM)** | **Nina (BM)** | **Niño (PB)** | **Timo (BM)** | **Vita (AMxSH)** | **All 5 Dogs** |
| **Prevalence (%)** | 10.6 | 10.6 | 10.3 | 10.5 | 10.5 | 10.5 |
| **n** | 834 | 834 | 836 | 839 | 841 | 4184 |
| **TP** | 86 | 81 | 84 | 86 | 83 | 420 |
| **TN** | 700 | 708 | 724 | 705 | 726 | 3563 |
| **FP** | 46 | 38 | 26 | 46 | 27 | 183 |
| **FN** | 2 | 7 | 2 | 2 | 5 | 18 |
| ***SEN %)*** | 97.7 [92.1-99.6] | 92.1 [92.1-99.6] | 97.7 [91.9-99.6] | 97.3 [92.1-99.6] | 94.3 [87.4-97.6] | 95.9 [93.6-97.4] |
| ***SPC (%)*** | 93.8 [91.9-95.4] | 94.9 [91.9-95.4] | 96.5 [95.0-97.6] | 93.9 [91.9-95.4] | 96.4 [94.8-97.5] | 95.1 [94.4-95.8] |
| ***PPV (%)*** | 65.2 [56.7-72.8] | 68.1 [56.7-72.8] | 76.4 [67.6-83.3] | 65.2 [56.7-72.8] | 75.5 [66.6-82.6] | 69.7 [65.9-73.2] |
| ***NPV (%)*** | 99.7 [99.0-100] | 99 [99.0-100] | 99.7 [99.0-100] | 99.7 [99.0-100] | 99.3 [98.4-99.7] | 99.5 [99.2-99.7] |
| ***ACC (%)*** | 94.2 | 94.6 | 96.7 | 94.3 | 96.2 | 95.2 |
| ***LR*** | 15.9 | 18.1 | 28.2 | 16.0 | 26.3 | 19.6 |
| **P** | <0.0001 | <0.0001 | <0.0001 | <0.0001 | <0.0001 | <0.0001 |

*BM: Belgian malinois; PB: Pit bull; AmxSH: Alaskan malamute by Siberian husky first generation cross.

**S5 Table. Phase 4: effectiveness assay to determine dog performance during in vivo screening under real-life conditions.** Performance metrics of three dogs in the Metro System of Medellin. Without new training or environmental habituation, dogs scent-interrogated 550 volunteers recruited on site. Simultaneous rRT-PCR in saliva led to detection of 17 COVID-19 patients, all asymptomatic or mildly symptomatic. Obviously, the research team was blinded regarding the diagnosis of all subjects. P values (Fisher’s Exact Test) express the probability that the dogs identified the patients infected by SARS-CoV-2 by chance alone.

| **Metric** | **Dog Name (breed*) and Effect Size of each Performance Metric [95% Confidence Intervals]** | | | | | | | | | | | |
| --- | --- | --- | --- | --- | --- | --- | --- | --- | --- | --- | --- | --- |
|  | **Andromeda (BM)** | | | **Niño (PB)** | | | **Vita (AMxSH)** | | | **All 3 Dogs** | | |
|  | **Effect Size** | **95% C.I.** | | **Effect Size** | **95% C.I.** | | **Effect Size** | **95% C.I.** | | **Effect Size** | **95% C.I.** | |
| **Prevalence (%)** | 3.1 |  |  | 3.1 |  |  | 3.1 |  |  | 3.1 |  |  |
| **n** | 550 |  |  | 550 |  |  | 550 |  |  | 1650 |  |  |
| **TP** | 15 |  |  | 6 |  |  | 14 |  |  | 35 |  |  |
| **TN** | 483 |  |  | 521 |  |  | 506 |  |  | 1510 |  |  |
| **FP** | 50 |  |  | 12 |  |  | 27 |  |  | 89 |  |  |
| **FN** | 2 |  |  | 11 |  |  | 3 |  |  | 16 |  |  |
| ***SEN %)*** | 88.24 | 65.7 to 97.9 | | 35.3 | 17.3 to 58.7 | | 82.4 | 59.0 to 93.8 | | 68.6 | 55.0 to 79.7 | |
| ***SPC (%)*** | 90.62 | 87.9 to 92.8 | | 97.7 | 96.1 to 98.7 | | 94.9 | 92.7 to 96.5 | | 94.4 | 93.2 to 95.5 | |
| ***PPV (%)*** | 23.08 | 14.5 to 34.6 | | 33.3 | 16.3 to 56.3 | | 34.1 | 21.6 to 49.5 | | 28.2 | 21.1 to 36.7 | |
| ***NPV (%)*** | 99.59 | 98.5 to 99.9 | | 97.9 | 96.3 to 98.8 | | 99.4 | 98.3 to 99.8 | | 99.0 | 98.3 to 99.4 | |
| ***ACC (%)*** | 90.6 |  |  | 95.8 |  |  | 94.5 |  |  | 93.6 |  |  |
| ***LR*** | 9.41 |  |  | 15.7 |  |  | 16.3 |  |  | 12.3 |  |  |
| **P** | <0.0001 |  |  | <0.0001 |  |  | <0.0001 |  |  | <0.0001 |  |  |

*BM: Belgian malinois; PB: Pit bull; AMxSH: Alaskan malamute by Siberian husky first generation cross.

## **S6 Table. In vitro determination of the limit of detection of SARS-CoV-2 by 4 canines.**

| **DOG** | **Patient Code** | **Viral Load (copies ssRNA/mL)** | **Limit of Detection (copies ssRNA/mL)*** | **Mean Limit of Detection (copies ssRNA/mL)** | **Standard Deviation (copies ssRNA/mL)** |
| --- | --- | --- | --- | --- | --- |
|  |  |  |  |  |  |
| **Andromeda** | 1 | 475.2 | ≤0.000000000004752 | ≤1.62E-12 | ±2.09E-12 |
|  | 2 | 47.2 | ≤0.000000000000472 |  |  |
|  | 3 | 47.2 | ≤0.000000000000472 |  |  |
|  | 4 | 79.2 | ≤0.000000000000792 |  |  |
| **Nina** | 1 | 475.2 | ≤0.000000000004752 | ≤1.62E-12 | ±2.09E-12 |
|  | 2 | 47.2 | ≤0.000000000000472 |  |  |
|  | 3 | 47.2 | ≤0.000000000000472 |  |  |
|  | 4 | 79.2 | ≤0.000000000000792 |  |  |
| **Vika** | 1 | 475.2 | ≤0.000000000004752 | ≤1.62E-12 | ±2.09E-12 |
|  | 2 | 47.2 | ≤0.000000000000472 |  |  |
|  | 3 | 47.2 | ≤0.000000000000472 |  |  |
|  | 4 | 79.2 | ≤0.000000000000792 |  |  |
| **Vita** | 1 | 475.2 | ≤0.000000000004752 | ≤2.61E-12 | ±3.03E-12 |
|  | 2 | 47.2 | ≤0.000000000000472 |  |  |
|  | 3 | 47.2 | Anosmia (estrus cycle) | NA | NA |
|  | 4 | 79.2 | Anosmia (estrus cycle) | NA | NA |

*All values for limits of detection appear with the symbol for “equal or less than” because we could not dilute the viral samples enough to obtain an exact result. All dogs were able to detect SARS-CoV-2 after 15 serial log10 dilutions of the saliva sample provided by each of four patients.

**S7 Table. Biosafety data for dogs, trainers, and physicians involved in sampling, experimentation, and medical care of COVID-19 patients.** Saliva sampling for rRT-PCR was performed twice for canine and human subjects at the end of phases 2 and 3 of the study. Despite heavy exposure of the dogs to COVID-19 patients with high viral loads, no one became sick or gave a positive rRT-PCR result.

| **K9 Team Member** | **rRT-PCR Result After** | |
| --- | --- | --- |
|  | **Phase 2** | **Phase 3** |
| Andromeda | Negative | Negative |
| Nina | Negative | Negative |
| Niño | Negative | Negative |
| Timo | Negative | Negative |
| Vika | Negative | Negative |
| Vita | Negative | Negative |
| Trainer-1 | Negative | Negative |
| Trainer-2 | Negative | Negative |
| Trainer-3 | Negative | Negative |
| Trainer-4 | Negative | Negative |
| Physician-1 | Negative | Negative |
| Physician-2 | Negative | Negative |

**S8 Table. Biosafety data. Testing the devices used to contain SARS-CoV-2 specimens.** After testing negative for SARS-CoV-2 in saliva, 5 groups of 3 golden Syrian hamsters (*Mesocrisetus auratus*) each were exposed during 4 days to SARS-CoV-2 directly (Group B, virus control) or enclosed in devices 1 (D1) and 2 (D2). Animals in test groups 1 and 2 were allowed to smell their devices but could not touch them, while the hamsters allocated to control groups A, B, and C could smell, touch, play, lick, bite, or eat the containment fabric.

| **Golden Syrian Hamster #** | **rRT-PCR result before exposure to SARS-CoV-2** | **Syrian Hamster Group** | **rRT-PCR Result after 4 days exposure to SARS-CoV-2** | | | | |
| --- | --- | --- | --- | --- | --- | --- | --- |
|  |  |  | **Experimental Arm** | | **Control Arm** | | |
|  |  |  | Device 1 | Device 2 | Device 1 | Virus | Device 2 |
| 1 | Negative | Group 1: D1 test | Negative |  |  |  |  |
| 2 | Negative |  | Negative |  |  |  |  |
| 3 | Negative |  | Negative |  |  |  |  |
| 4 | Negative | Group 2: D2 test |  | Negative |  |  |  |
| 5 | Negative |  |  | Negative |  |  |  |
| 6 | Negative |  |  | Negative |  |  |  |
| 7 | Negative | Group A: D1 control |  |  | Negative |  |  |
| 8 | Negative |  |  |  | Negative |  |  |
| 9 | Negative |  |  |  | Negative |  |  |
| 10 | Negative | Group B: virus control |  |  |  | Negative |  |
| 11 | Negative |  |  |  |  | Negative |  |
| 12 | Negative |  |  |  |  | Positive |  |
| 13 | Negative | Group C: D2 control |  |  |  |  | Positive |
| 14 | Negative |  |  |  |  |  | Positive |
| 15 | Negative |  |  |  |  |  | Positive |
