# Supplementary material for: Highly sensitive scent-detection of COVID-19 patients in vivo by trained dogs
Source: PLoS One. 2021 Sep 29;16(9):e0257474. doi: 10.1371/journal.pone.0257474 (PMC8480816; doi:10.1371/journal.pone.0257474)
Supplement: Attachment — Submitted filename: Response_to_Reviewers.docx [file pone.0257474.s002.docx]

Medellin, 29 July 2021.

We are indebted to the Reviewers forever. Their comments, questions, and suggestion showed us that we included a great deal of repetitive information while leaving many key areas not addressed at all. Our point-by-point answers appear below, immediately after each issue raised by Reviewer 1 (in blue font).

**Reviewers' comments:**

**Comments to the Author**

- Reviewer #1: Since the outbreak of COVID-19 pandemic several peer-reviewed papers have been published, showing that dogs, even after a relatively short operant conditioning training, are able to discriminate odor samples collected from COVID-19 positive donors, from those collected from healthy controls. The studies demonstrated surprisingly high sensitivity and specificity >90% of COVID-19 detection by trained canines.
  The general aim of these studies is to find a simple, inexpensive and high throughput screening method for detection of both symptomatic and asymptomatic humans infected with SARS-CoV-2 virus, to isolate such people to prevent further dissemination of the virus, even without being aware.
  The reviewed paper generally supports previous findings, however, it includes some interesting issues that have not been addressed in previously published papers, as well as some novel methodological approaches. Therefore, this paper has some merits that makes it worth publishing.
  In my review I will focus chiefly on canine aspects of the study. Not being an epidemiologist, I feel not competent enough to evaluate the bio-safety and preventive measures to avoid contagion with SARS-CoV-2 during these experiments and to evaluate methods like rRT-PCR assay and RNA quantification to assess contagion hazard during collecting and handling of samples, as well as during in vitro and in vivo tests, as presented in the attached videos. However, in view of the latest news on new mutations of the virus, which are supposed to be more infectious that the previous variants, the procedure demonstrated on video S2 seems to neglect one of the main preventive measures that are recommended at least during the first phase of the pandemic, and namely distancing, avoiding direct contact e.g. by shaking hands, disinfection etc. Although the authors showed that the dogs and the persons involved in the experiments did not contracted the virus, demonstrating that nothing had happened at disobeying the preventive recommendations, does not mean that these recommendations can be generally disobeyed.
  In my opinion, before accepting this manuscript for publication the structure of the submission should be improved, since it is chaotic, making the paper too long and difficult to follow. The authors divided the description of the methodology and results into main body of the paper and supplementary information. It is not clear to me why the authors placed a part of the description of the material and methodology in the main body of the submission and another part in the supporting information. The authors in the supporting info reiterate some details that were given in the main body of the submission. As supplementary info I would rather expect detailed raw data, video files (which are in fact attached, however, some short explanations to the details shown on the videos would be helpful), instead of a longer text with methodology, results, acknowledgements and references. It is impossible to change a culture in a year. Colombian people like proximity and casualness, and it is still evident despite the pandemic. That said, we did not shake hands with a patient or among us, always used alcohol to eliminate virus from our gloved hands after coming in contact with a sample from any individual, and tried to keep social distance when it was possible. The experiment in the Metro System of Medellin was a real-life approach to what the screening work would be for dogs and scientists, and it is what S2 Video shows. The paper was reviewed to lessen the chaos, increase clarity, and to shorten contents as much as possible, not only avoiding repetitive wording, but moving all details of the Methods to the supporting information file. Now, Methods are short, clear and concise, but all the details are available in the supporting information. We also added to this version the required explanations to the videos.

The authors listed 5 aims of their study
Aim #1 - Can dog breeds other than working breeds commonly used for olfactory detection, in this case pit-bull and nordic mix sled-dog (siberian husky x alaskan malamute), be trained and employed as medical detectors. However, using only one single dog of a breed does not tell much about ability of the breed to be efficiently trained for a specific olfactory task. In almost every dog breed there are individuals that can be successfully trained to perform any task with reasonable training workload and within reasonable time-frame, but it does not mean that a breed is generally recommended for the task. Therefore, I suggest to discuss some limitation of this aim in the Discussion section. Absolutely. We were not clear in describing this objective. Now, a modified wording intends to convey this message: individual dogs with certain attributes that enhance their working abilities are suitable for scent-detection training, independently of their breed.

Aim #2, assessing how is the lowest number of pattern odor donors for the training to achieve generalization of odor signature characteristic for COVID-19 in order to detect target odor in human population. It is generally recommended that dogs trained to detect diseases in humans should be trained on a number of pattern odor samples (high number of donors with a disease diagnosed). It is supposed that dogs could memorize individual components of odor samples (individual human odor) during training, if samples from too few donors are used, and then may have problems, when it comes to detection of odor samples collected from other donors. This issue is particularly important in COVID-19 detection due to high contagiousness if the SARS-CoV-2 virus and still not knowing exactly how people can contract the virus, especially with regards to new virus mutations. This would mean that the less COVID-19 positive donors from whom the samples have to be taken for the dog training, the lesser hazard to contract the virus. The authors demonstrated that pattern samples taken from only three donors, are sufficient to generalize the COVID-19 odor to the other nine people. The main question is, however, to what odor the dogs really alert. Previous studies showed that the dogs can be trained to alert to sweat samples (Grandjean et al. 2020, Angeletti et al. 2021) or to saliva or tracheobronchial secretions (Jendrny et al. 2020) collected from COVID-19 positive donors. As to the human sweat, it is commonly acknowledged that it contains individual odor component, genetically determined as well as influenced by bacterial decomposition. To my knowledge, no exact data are available as to the individual component of the odor of the saliva or tracheobronchial secretion. If the dogs almost perfectly generalize the sweat or saliva/tracheobronchial secretion odors of only three COVID-positive donors, this would mean that there is a defined, dominating odor of one or a single combination of few volatile organic compounds that masks the individual human odor. If ethyl butanoate was found as the most abundant VOC in the breath COVID-positive patients, (Chen et al. 2020), the question is if ethyl butanoate could be considered a main candidate for a COVID-19 odor marker. Usually, however, it is a variety of combinations of VOCs in human odor samples, and as it was shown in studies on cancer odor markers, no single odor marker of the disease exists. The issue of the origin of the hypothetical COVID odor should be addressed in the Discussion section. We acknowledge the importance of identifying the VOC constituting the scent-print of COVID-19, and tried to find an answer to support and intelligent argument in our Discussion section. Unfortunately, the data available is contradictory and blurred. Chen et al (reference 22 in our manuscript), using gas chromatography-ion mobility spectrometry (GC-IMS) were first proposing ethyl butanoate as the single most important VOC in COVID-19 patients, but their findings were not reproduced by Grassin-Delyle et al (ref. 23), who but recurred to gas chromatography-mass spectrometry (GC-MS) to find four VOC (methylpent-2-enal, 2,4-octadiene, 1-chloroheptane, and nonanal). Later on, Ruszkiewicz et al ([https://doi.org/10.1016/j.eclinm.2020.100609](https://doi.org/10.1016/j.eclinm.2020.100609)), using GC-IMS, found ethanal, octanal, acetone, butanone, and methanol. None cites or discusses the other’s findings. More recently, Nurputra et al (<https://doi.org/10.21203/rs.3.rs-750988/v1>) recurred to an artificial intelligence algorithm to decide which of the many VOC was diagnostic of COVID-19. They discuss the reasons behind the different publications concluding that, besides methodology, people around the world might have VOC for COVID-19 that change depending on their microbiota and external environments.

What we found was that results from gas chromatography coupled to mass spectrometry vs. ion mobility spectrometry are not comparable. The fact is that dogs do identify easily the scent-print of COVID-19, whatever it might be, and they have been successful in all continents. Recently, one of us peer-reviewed a manuscript in which the authors compared the rate of success of trained canines detecting COVID-19 in saliva, sweat and urine. Their dogs excelled in all three specimens without additional training. It suggests that the VOCs emitted by COVID-19 patients are well defined for the dogs and detected everywhere in the anatomy of the subject. It includes, besides physiological secretions, the skin itself and the air exhaled by the patient, as our dogs showed (they often insisted in smelling the nose despite the mask when their subjects were lying down or bending over). Instead of speculating in the Discussion about the VOC of COVID-19, we cited in the Introduction the works based on the soundest methodology (refs. 22 & 23).

Aim #3, assessing the effects of sample numbers on the diagnostic metrics in vitro and in vivo under controlled experimental conditions, i.e., efficacy. Assessing such parameters as detection sensitivity, specificity and accuracy is a typical aim in such type of studies, allowing comparison and/or confirmation or throwing into question the results of other studies. The authors of the reviewed article include additional detection parameters like PPV, NPV and prevalence, which were not considered in any of the previous papers. We modified the Discussion to, among several other reasons, explain the meaning and importance of predictive values when validating a diagnostic or screening test.

Aim #4 - Quantitative assessment of the detection threshold in terms of copies of single stranded viral RNA per milliliter (ssRNA/mL). This data would be helpful in collecting and preparing samples for canine training and testing. However, a question arises if the dogs alert to viral RNA as such, which is probably odorless, or to products of the changed cell metabolisms caused by the virus. Further question is if a given number of copies of ssRNA/ml is directly related to the amount of a single VOC or VOCs produced, to which the dogs probably alert. This point is discussed below in the answer to several question of R1.

Aim #5 - assessing real-life performance of the dogs in in vivo screening COVID positive passengers was an original and to my knowledge not addressed aim in previous studies on medical detection dogs trained to detect diseases in humans.
The in vitro part of the study uses experimental setup that differs from previously published studies both on cancer markers detection and COVID-19 detection by trained canines. The authors conducted the trials with dogs not indoors as it was the case in all other studies known to me, but outdoors, arranging 100 odor samples in 10 rows of 10 samples 2 m apart. This setup requires an area of 400 square meters, which may be not always available for experimentations. Secondly, conducting trials outdoors involves such hardly controllable confounding factors as distraction of dogs, weather conditions, uncontrollable migration of odor plume etc.
The videos attached to the supplementary info, show apparently a training phase under single-blind protocol meaning that the handler was blind to the position of the target sample in the lineup or to the COVID-positive person in the queue, but the experimenter who activated the clicker to give a signal to the handler that the alert was correct, was aware of the position of the target. While on the video S1 all alerts of the dog were rewarded, and the behavior of the dog, the handler and partly the experimenter can be seen, the video S2 was recorded probably for the purpose of the TV, including some shots from different positions that are impressive for the TV audience but does not document well the behavior of the dog, the handler and the experimenter. In the video S2 the dogs are sometimes rewarded after alert and sometimes not. Thus, it is not clear whether the video S2 shows a single blind training phase and alerts that are not rewarded were false, or it shows a real screening and the not rewarded alerts were true double-blind trials. It would be useful to have some comments of the authors in the supplementary info on particular alerts and rewarding of dogs recorded on the videos. There is a basic difference to a real screening which is a true double-blind procedure, meaning that nobody knows if the alert was correct or false, and the dog should not be rewarded for a false alert. The dogs in this study are working on leash, which could be justified by a possibility to urge the dogs to work systematically i.e. sniffing all 100 in vitro samples and to sniff systematically people on the subway station. Although it cannot be seen that the handler gives overt cue to the dogs, and the dogs are well trained to work systematically, however, the leash is considered by many trainers a wired communication between handler and dog and is questionable in this type of detection. On the video S1 the experimenter with the clicker follows closely the dog in the scent lineup. Even if the dog does not seem to pay attention to the experimenter at this stage of deployment, it could be only a matter of time for the dog to learn to observe the experimenter to decipher when it should alert or not (the „Clever Hans effect”). Although theoretically there could be a variable reinforcement ratio, meaning that animals can be rewarded not after each correct behavioral response, but only after some, in practice the dogs, especially after a longer deployment, when not being rewarded frequently may try to earn a reward by using a trial-and-error strategy, which causes making more false alerts. The problem with detection dogs is that they work not to detect odors which have no biological relevance or rewarding values for canines, but the dogs rather work to find an opportunity to earn a reward, due to association between trained odor and a reward, that was created in the process of operant conditioning. Therefore, the detection sensitivity and specificity may vary during the deployment period and should be systematically checked. At each of the training or deployment period an appropriate „success rate” should be settled to maintain dogs’ interest for work. While during the real screening test in the lineup of 100 samples it can be controlled how many samples are of known status (positive and negative) and how many samples are of unknown status, when testing people in vivo in a queue, it is hardly to control who of them are true positive, true negative to reward/not reward the dog for a correct response, unless such persons diagnosed previously are available for the trials. These issues should be addressed in the Discussion section.
If the dogs in this study did not mark as positive any of the patients with respiratory diseases other than COVID-19 this would mean that a very specific VOC or combination of VOCs are characteristic for COVID-19 and should be identified chemically. The other question is if the same VOC or VOCs are characteristic for any new mutations of the virus. This issue should be addressed in the Discussion. Secondly, if the dogs, independently of the experimental design (in vitro and in vivo) and independently of COVID-19 severity, ranging from asymptomatic to pre-symptomatic, sick and very sick patients, alerted with a very high accuracy, the question should be discussed to what they actually alert and what is the origin of the odor and how the odor will be produced. S1 Video shows an efficacy experiment in vitro after phase 2 training, in which the handler was blind but not the director of training, who remains behind the dog-trainer duo to avoid giving them any subjective clue about the position of the positive stimuli. Video S2 shows a real-life (effectiveness) experiment, therefore the COVID-19 status of the population under screening was unknown to all scientists and participants. To decide when to reward the dog, the director of training judged if the dog was displaying the body signals that are typical of a true positive finding. However, rRT-PCR results showed afterwards that this approach caused a high rate of mistakes in rewarding, confusing the dogs and affecting negatively their performance (false-positive alerts increased dramatically). We are testing a new approach to alert training and rewarding that avoids this problem and allows unleashing the dogs, but have not been able to obtain a grant. A comment was added to each video addressing this and other points highlighted by R1, and the blind protocol was made clear for each experimental phase in Methods. The Discussion now has a paragraph focused on the reasons for the high number of false alerts during the effectiveness assay in the Metro System.

- Minor remarks

L.36 and elsewhere „interrogating” is not a good wording, I suggest „sniffing”. Most uses of the interrogation verb with that meaning were changed to sniff.

L.36 there are some doubts if sniffing directly the body of patients would be an ideal method because there are some drawbacks e.g. interactions between subjects and dog, fear of dogs, refusal to be sniffed etc. Make a brief discussion about the advantages and disadvantages. This point was included in the Discussion.

L.56 it could be expected that dogs that are trained on saliva samples would make more false alerts when sniffing hand palms. We did not train our dogs to sniff the hands, they all went for the hands spontaneously because their first exposure to humans with COVID-19 took place in bed-ridden patients whose hands are the first part of the body that the dogs had access to. However, we did not worry about it because they do sniff the whole body of most patients and even their belongings and surroundings if necessary to locate the source of the target scent.

L.103 Here the study objectives and aims should be placed. The whole paragraph dealing with Study Objectives is now located before the last paragraph of the Introduction section.

L.115 „For maximal output” - this wording is not clear: working dogs have to be rewarded not for a maximal output but to produce an association between an odor and the reward (operant conditioning). Correct. The expression “For maximal outcome” was eliminated from that sentence. Now it reads: “Working dogs must be rewarded for each positive finding with a prize that conveys an extremely high value for them, and performance depends heavily on the intensity of the expectations that such reward generates in their brains during foundational training [25,26].”.

L.115 „….dogs must be rewarded for each positive finding….” – not quite precise statement: there is also a variable reinforcement ratio – rewording not for every bet only for some of the correct positive findings. This issue concerns real screening under „true” double blind conditions, where the dogs should not be rewarded for each positive finding because it is not known if the alert was correct or false. That is correct. However, during the foundational phases of training, a schedule of continuous reward is absolutely essential to teach the dog that every and all correct associations will always represent a great reward for him. To adhere to R1 suggestion, we added the word “foundational” at the end of the sentence.

L.121 Rather „olfactory ability” than „scent power”. The expression “scent power” was changed for “olfactory ability”.

L.147 Do the do the dogs identify the virus or alert to the VOCs produced by changed cell metabolisms during infection ? The virus itself might not emit an odor at all, but the infection process that takes place when a human subject has COVID-19 undoubtedly emits a specific, unique odor, the scent-print of the disease that we trained the dogs to identify. To be consistent with that principle, we changed the sentence this way: “In order to produce dogs capable of screening humans for SARS-CoV-2 infection, teaching them to identify in vitro the scent-print of COVID-19 was a mandatory prerequisite.”.

Tab.1. were some SARS-positive donors asymptomatic ? No. All donors of the first 12 respiratory secretion specimens were very sick COVID-19 patients, as established in lines 148 and 149 of the submitted manuscript, and in the heading of column 5 of Table 1, which reads “Days Sick”. Control subjects 13-112 were healthy people with negative rRT-PCR for SARS-CoV-2 (last row of Table 1).

L.155 which environmental modifications are meant ? We added this sentence to answer that question: “Phase 1 (in vitro recognition) lasted 28 days during which we trained the dogs to recognize in vitro the scent-print of SARS-CoV-2 under a wide variety of environmental modifications that included but were not limited to time of each day, weather, training field, altitude above the level of the sea, distraction and hiding devices, age and temperature of the target samples, noise level and origin, distracting smells, training time (1 to 60 minutes), rewards, dog collars and leashes, etc. The only aspects of training that remained constant during phase 1 were the positive specimens from the first three donors in Table 1 (one from each hospital), the use of 0.9% sterile saline solution as negative controls, the constant schedule of reinforcement, and the dog-trainer duos.”.

L.166 – what was the correct behavior (alert), who activated the clicker ? The paragraph was edited this way to answer both questions: “The director of training (AFVJ) pressed a clicker device to mark the correct behavior (i.e., lying down to alert on the identification of the SARS-CoV-2 specimens), and the trainer immediately rewarded the dog, who knew beforehand that the click always was followed by a reward.”.

L.169 how many trials per day ? It varied as training progressed, but the longest training sessions had 1 hour of work for 2 hours of resting. When dogs worked the hardest, they did it 3 hours per day (interspaced with the corresponding 2h resting periods). This sentence was added: “Training sessions varied from 1 to 60 minutes followed by resting twice that time.”.

L.173 – usually dogs make some false alerts of misses from time to time. How many error-free consecutive trials have to be made? This sentence was added: “(zero errors in a 10-sample field during 10 repetitions varying the target prevalence).”.

L.194 what was a criterion for passing to the next training stage (% of correct alerts?) This paragraph was modified so the answer to the last two questions could be found in the same sentence.

L.198 The training method for "in vivo” screening was not sufficiently described. How many donors were used for the training? Here it could be understood that 3 donors were used for the training, however in supplementary info page 5 third line from the bottom 400 subjects used for the training are mentioned. What was the first stage of the in vivo training ? This paragraph was modified to include the answer: “Phase 3 of training (in vivo screening) took 56 days during which the dogs learned to identify COVID-19 patients by scenting the human body. Each canine was trained with 400 subjects who did not participate in the experiments, 100 hospitalized patients (COVID-19 prevalence: 40%) and 300 HCW (COVID-19 prevalence: 7%). Most of the time in phase 3 training was dedicated to improve specificity and positive predictive value, because sensitivity and negative predictive value never represented an obstacle. The focus of the study was in vivo screening; in vitro training and experimentation were designed as a necessary pre-requisite to attain the main goal, the canine detection of COVID-19 by direct scent of the human body.”.

L.201-202 – what kind of samples were collected for the in vivo training (sweat or saliva or secretions) ? were those samples taken only from 51 COVID positive patients, meaning that >100 samples per donor ? How were the samples handled for biosecurity ? In vivo training did not involve sampling because the objective was to sniff the human body directly. However, we did use during training (not for in vivo experiments) saliva from 300 health-care workers (COVID-19 prevalence: 7%) to make sure that the dogs were not fixed on some odoriferous clue from the hospital environment. HUSVF has strict and specific biosafety rules that we adhered to carefully depending on each patient condition: general isolation (applied to any patient), drops isolation, contact isolation, and airborne transmission isolation. Clinical specimens were handled always under a biosafety class III laminar flow cabinet. We eliminated from this paragraph the mention of the 51 patients because it was confusing (they were COVID-19 patients who participated in phase 3 experiments but were diagnosed by antigen testing, and are mentioned now only in the Results section).

L.207 - dog training was partly described in the paragraph Design and Sample Size. There is a confusion in describing particular sections: first the animals should be described, then the collecting of odor samples, dog training and statistical methods. This time we took care of organizing better the narrative of the manuscript.

L.226-227 „..only after obtaining very high diagnostic metrics in vitro…”. – how high were these metrics ? >90% ? This question was answered above (L.173).

L.229 – sniffing hand palms without prior washing may involve such issues as confounding odors of food, when a person ate something shortly before being sniffed by the dog, attractive or aversive individual odor of some persons, etc. Also, dog-human interaction, fear of dogs may play a role. These issues should be discussed in the revision. We trained the dogs with a variety of distracting odors including different types of food items. It never represented a problem in our approach. However, we did have 4 subjects (all positive) who rejected the dog interrogation due to fear. The point was included in the Discussion.

L.320-322 Does the RNA smell? – probably dogs alert to some VOCs produced by cells during infection with SARS-Cov-2. Is there a direct relation between the number of copies of ssRNA/mL and the amount of those putative VOCs ? Based on the available evidence regarding scent theory, dogs should alert on a specific mix of VOCs emitted by COVID-19 patients. Interestingly, when in doubt, the dogs always tried to sniff the breath of the patient as exhaled directly from nose and mouth. This sentence was added to that section: “Since we did not determine the nature and relative concentrations of the VOCs of COVID-19 patients, the only method available to quantify the LOD was the RNA concentration of SARS-CoV-2 per mL of saliva, which is very precise. It does not mean that RNA has odor, but provides a quantitative approach to the actual potency of canine olfactory system to detect the scent-print of COVID-19.”.

L.337 Detailed methodology should be described in the M&M section of the manuscript and not in the Results. The mentioned sentence was eliminated; it had been placed there by mistake.

L.341 and 351. In practice it is hardly possible to achieve all trials without any error. Only a series of error-free trials is usually possible. The question is how low was such series – one or 5 or 10 or more trials on 100 samples ? We did not train the dogs on fields with more than 10 samples because the design called for 100-sample fields ONLY for experiments. That way we were sure that the dogs were not expecting a reward after ending each 10-sample row. During training, the largest series used a 10-sample field, and we asked for zero errors in 10 repetitions varying the prevalence and the location of the target scent. This point was already addressed in the answer to questions in Line 173.

L.352 – were „zero” trials conducted ? (with no target odor among the 100 negative samples). No. However, we did many training sessions with zero prevalence among 10-sample fields. We did not run experiments with zero prevalence in the experimental 100-sample field because the pandemic prevalence in Colombia was already greater than 1% when we started in vivo experiments. Finally, it should be mentioned that in vivo experiments in the Governor’s Building included 320 subjects, 4 infected with SARS-CoV-2, but none of these 4 infected patients accepted the dog interrogation. It meant for the dogs to scent 316 negative individuals. The dogs performed well with the first 100, but we had to include a “positive” decoy after that because it was evident that the dogs were becoming frustrated. Positive decoys allowed us to provide a positive stimulus to reward the dogs when prevalence approached 0%, were not part of the data (of course), and were impersonated by a volunteer hiding a D2 device with a fresh saliva specimen from one of the 4 COVID-19 patients who refused the dog test.

L.369-370 „…When the dogs interrogated the first positive patient, all six recognized the scent-print of SARS-CoV-2 and went down without hesitation….” - on videos S1 and S2 played at slow motion some hesitations of dogs can be seen. The first positive patient could be actually alerted without any hesitation but the question is if the further (many) patients would be indicated without any hesitation as well. That sentence refers only to the first of all positive patients ever interrogated by scent by our dogs. In general, dogs did not hesitate to lie down during an alert, but it was not always the case. Based on this observation from R1, we eliminated that sentence from the manuscript.

L.370-373 „….it proved to be a difficult endeavor…..”. It seems that the training of dogs to alert to SARS-Cov-2 on any belongings of the patients would be too challenging. It would be better to standardize the method and to improve canine proficiency at working on unified odor samples or on people. We eliminated that sentence from the manuscript without avoiding the discussion of the point raised by R1. However, we feel that in vivo screening for pathogens is a very promising approach to prevent transmission to innocent bystanders, like hospital-acquired infections (HAI), that kill 99,000 humans every year in the US only. The main source of HAI are the other patients, and the main mode of transmission is contact of health-care workers with the colonized or infected patient followed by contact transmission to the next patient. The key to stop this chain of contagion is a very rapid identification of all patients harboring the pathogen, and a trained dog can detect that faster and much cheaper than any other available screening method. It is true that training for discrimination between colonization and infection is challenging in the case of SARS-CoV-2, but it will not be for HAI because these pathogens usually colonize the sources without infecting them. Therefore, a dog that detects environmental or patient colonization would be an advantage. It does not mean that improving canine proficiency screening for COVID-19 is not desirable or achievable, it means that the potential uses for screening dogs are many and very useful for humanity. The same was already demonstrated by USDA scientists with dogs trained to screen for plant pathogens (Gottwald et al, references 20 & 21 in the manuscript): they saved millions to the agricultural industry. Applied to humans, dogs would not only save money, they would save many lives.

L.377 It seems that the confounding effect of individual odor of positive patients tested in vivo plays no role ? The data suggest that there is no confounding effect of the individual odor of the positive patients. The main argument is the large difference in *PPV* between efficacy (70%) and effectiveness (28%) trials in vivo. The real difference between those two trials was that (1) dog reward was highly inaccurate from our part during the effectiveness assay; and (2) we had no way of controlling environmental contamination with COVID-19 odor in the public transportation system, while it was strictly controlled in the hospital and governors’ office. Both arguments are now part of the Discussion.

L.397-398 some parts of the description of material and methods are doubled and scattered throughout the main text and the supplementary info. This makes the whole submission too long and difficult to follow. In the new version we did our best to correct this particular problem. Now, there is no repetition of information, and only a brief summary of the Methods are in the Manuscript, leaving all details for the Support Information file.

Figs 5 and 6 seem to be redundant if the same results were given in tables 3 and 4 respectively ? In the new version, only the figures are part of the Manuscript, while the tables showing the data belonging to those 2 figures are part of the Support Information file.

L.537-542 „…We also observed several times during training that the dogs spontaneously marked as positive the scientists that had touched any COVID-19 patient, or the cell phones of nurses and physicians in care of COVID-19 patients. It means that trained canines detect the scent-print of SARS-CoV-2 in contaminated individuals or in their belongings and, since contamination could lead to infection [39], the dogs actually identify potential COVID-19 cases before infection takes place…” – this statement should be critically revised and clear limitation should be indicated. If the dogs „several times” alerted positively to the experimenters or to the belongings of the hospital staff, there is no proof that the dogs identify COVID-19 before infection takes place. This could be simply false alerts and there are no proofs how many misses (false negative = not detecting cases before infection) would be found. We stand corrected. The new version does not have such speculative remark. To address the point, we dedicated a paragraph of the Discussion to it.

L.569-573 „…..expert detection dogs remembered a new scent-print 98% of the time as long as it was located first in a line-up with five distractors, but performance went down when the positive stimulus was located farther, dropping to 11.5% at the sixth location [28]. Therefore, it seems impossible for a dog to remember the odor of up to 12 different individuals randomly allocated among 100 distractors….” - the other studies do not support this finding, as the dogs are able to memorize much more than 12 individual scents and the percentage of alerts to memorized scent does not depend of the location in the lineup. We could not find any solid evidence of dogs being able to remember after just a second of contact any number of patients, let alone more than a dozen. However, we changed the argument using our own data to demonstrate that the dogs were not using memory to detect COVID-19 patients. It is much stronger now.

Fig 7. Testing 550 individuals in vivo within 5 hours seems to be definitely too strenuous for dogs. Even testing 110 persons within one hour of work, depending on weather conditions would be very strenuous. It should precise how long was a work bout without break. The actual experiment took two days. We tested 250 patients the first day and 200 the second day. Each dog worked two sessions of one hour each day. On average, the dogs screened 1 patient every 5 seconds, 12/min, 720/h. Counting the time spent by the 3 trainers rewarding their dogs and moving to the next patient, screening went at a rate of 1 subject every 30 seconds, 2/min, 120/h.

In summary, this paper has some merits and would be acceptable for publication in PloS after major revision, considering all critical points and indicating clearly limitations of the method.

Our sincere appreciation goes to R1 for her/his patience and scientific integrity in the process of thoroughly reviewing and constructively criticizing this work.

**Omar Vesga, MD.**
