# Supplementary material for: Highly sensitive scent-detection of COVID-19 patients in vivo by trained dogs
Source: PLoS One. 2021 Sep 29;16(9):e0257474. doi: 10.1371/journal.pone.0257474 (PMC8480816; doi:10.1371/journal.pone.0257474)
Supplement: Attachment — Submitted filename: Response_to_Reviewers.docx [file pone.0257474.s003.docx]

Medellin, 27 August 2021

Dear Dr. Ito,

This document contains my point-by-point response to you and to Reviewer 1. We are indebted forever, because thanks to the peer review and editorial process, the manuscript is now readable and understandable.

In blue font, I left your words and those of R1. In red font, my answers. When citing the new text, I used quotes, but left the new text in black font to ease reading. The last step was to shorten the manuscript, and then it was reviewed by an English-native scientist familiar with the methodology (**Tonie E. Rocke, PhD. USGS National Wildlife Health Center**). Although all suggestions from R1 were not only welcomed but incorporated in the final version of the manuscript, a few might have been let out (unintentionally) or modified slightly along this process.

Sincerely,

Omar Vesga, MD.­­

­­­­­­­­________________________________________________________________________________

PONE-D-21-16873R1
Highly sensitive scent-detection of COVID-19 patients in vivo by trained dogs
PLOS ONE

Dear Dr. Vesga,

English should be edited as the reviewer suggested.

Done. After introducing the changes described below, the manuscript was reviewed and edited (in style) by a US native scientist who is familiar with the methods employed in this study.

Please submit your revised manuscript by Sep 18 2021 11:59PM.

Please include the following items when submitting your revised manuscript:

- A rebuttal letter that responds to each point raised by the academic editor and reviewer(s). You should upload this letter as a separate file labeled 'Response to Reviewers'.

Done (this document).

- A marked-up copy of your manuscript that highlights changes made to the original version. You should upload this as a separate file labeled 'Revised Manuscript with Track Changes'.

Done (uploaded along with this document).

- An unmarked version of your revised paper without tracked changes. You should upload this as a separate file labeled 'Manuscript'.

Done (uploaded along with this document).

Etsuro Ito
Academic Editor
PLOS ONE

Journal Requirements:

Please review your reference list to ensure that it is complete and correct. If you have cited papers that have been retracted, please include the rationale for doing so in the manuscript text, or remove these references and replace them with relevant current references. Any changes to the reference list should be mentioned in the rebuttal letter that accompanies your revised manuscript. If you need to cite a retracted article, indicate the article’s retracted status in the References list and also include a citation and full reference for the retraction notice.

References checked for completeness and correctness; additional references replaced some cited in the previous version. We found no retracted papers.

Reviewers' comments:

Reviewer's Responses to Questions

**Comments to the Author**

Reviewer #1: The authors addressed sufficiently my comments in their rebuttal, but have only partly incorporated amendments into the revision. First of all, the structure of the submission should be further improved, since it remains not concise enough and chaotic. Eventually, it is up to the Editor of PloS to decide if the overall structure of the revision is acceptable or should be changed. In my opinion, however, the main body of a paper should contain all essential information and data of the study. I have no comments to the revised Introduction section including hypotheses, except for the lines 141-145 that fit rather to the M&M section than to the Introduction. I think in the Material and Methods the reader should be able to find all essential information on animals, collecting and handling of odor samples, training procedure and statistical methods used in the study, without searching for supporting information. The supporting information is useful for somebody who wants to learn all additional details of the study. In the present form of the revision, the reviewer (and the readers), have to jump to the supporting info and back to the main text, which is inconvenient and makes following of the paper not easy.

We tried carefully to incorporate all the amendments suggested by R1. When in disagreement (once or twice), we explained our reasons (in this response).

Being not an English native speaker, I will not evaluate the language of the paper, however, to me the revision requires extensive editing of English by a native speaker, who is familiar with specific wording concerning both COVID-19 epidemiology and canine detection. Nevertheless, I try to give some suggestions.

The structure was edited by myself first, and then by a native English-speaking scientist, as suggested by R1, trying to make the manuscript concise and clear without sacrificing the substance. It moved the line numbers and I had to write here the text so R1 can check what we did with every one of her/his observations. The Results section was incorporated back to the manuscript. Now, there are no Methods in the Supporting information file.

L.48-49, I suggest "...A real-life (in vivo) performance was determined 75 days after in vitro effectiveness assay...”.

It was done as suggested, but correcting the expression underlined above. Now, the sentence reads this way: "Seventy-five days after finishing in vivo efficacy experiments, a real-life study (in vivo effectiveness) was executed among the riders of the Metro System of Medellin, deploying the human-canine teams without previous training or announcement.”.

L.51 Here and elsewhere: is the word "interrogation" appropriate in this context ? I would suggest: " ...Three dogs were used to examine the scent of 350 volunteers, who agreed to participate both in test with canines and in rRT PCR testing...”

It was done as suggested, correcting the expression underlined above. Now, the sentence reads this way: “Three dogs were used to examine the scent of 550 volunteers who agreed to participate both in test with canines and in rRT-PCR testing.”.

L.55-58 This statement is imprecise. The task of the dogs is not to discriminate odoriferous contamination from infection since probably all odor samples collected in reality, are to some extent contaminated, and no pure "infection odor" exists. The dog should be rather trained to ignore contamination and to indicate "infection odor" regardless its contamination.

The imprecise statement was eliminated from the Abstract. Now it reads this way: “Canine scent-detection in vivo is a highly accurate screening test for COVID-19, and it detects more than 99% of infected individuals independent of key variables, such as disease prevalence, time post-exposure, or presence of symptoms.”. Those variables had been mentioned before, so we just move them down without altering the meaning of the Abstract.

L.76 The nations did not demonstrate. I suggest ".. It was clearly demonstrated in several countries that....".

The new line reads this way: “It was clearly demonstrated in several countries that early and massive testing, followed by immediate isolation in designated areas away from home and rigorous contact-tracing, were the only measures that effectively stopped the pandemic even before the first vaccine was available [6].”.

L.111 I suggest „ … the dog must be reinforced……with a reward……”

The new line reads this way: “Working dogs must be reinforced for each positive finding with a reward that conveys an extremely high value for them, and performance depends heavily on the intensity of the expectations that such reward generates in their brains during foundational training [26].”

L. 115 I suggest „…selection process of dog candidates for the olfactory training e.g. for detection of explosives is needed….”

The new line reads this way: “Even within optimal training conditions, not all canine individuals will give their best to gain a reward, and a rigorous selection process of dog candidates for the olfactory training is needed [27].”.

L.117 „exceeds” instead of „excels”.

The new line reads this way: “Bloodhound is a working breed with exceptional olfactory ability,”.

L.118 „of working in the scent lineup”.

The new line reads this way: “…but is not suitable for medical detection because most individuals do not enjoy working in the scent lineup.”.

L.119 „human odor” instead of „human subject”.

Line 119 was eliminated.

L.123. „….learning ability, trainability and ability to cooperate with humans…” instead of „minds”.

The new line reads this way: “but cognition research is providing solid evidence that dogs indeed have unusual learning ability compared with other nonhuman animals…”.

L.141-144 – this passage fits rather to the M&M section than to the Introduction.

It is the last paragraph of the introduction, and as such it has the purpose of preparing the reader for the methods and results. It is the usual style in most journals focused on human medical sciences, including PLoS ONE, which states this in their Submission Guidelines under the topic “Introduction”:

The introduction should:

- Provide background that puts the manuscript into context and allows readers outside the field to understand the purpose and significance of the study
- Define the problem addressed and why it is important
- Include a brief review of the key literature
- Note any relevant controversies or disagreements in the field
- Conclude with a brief statement of the overall aim of the work and a comment about whether that aim was achieved

L.145 „Ultimate goal” instead of „product” (?).

The intention of the sentence is to confirme that the aim was achieved. Therefore, there is no place to add an additional goal when all five objectives had been mentioned in the previous paragraph of the introduction. Since the noun “product” (*a thing produced by labor*) is what R1 objects, we changed it for “outcome” (*a final product or end result; a conclusion reached through a process of logical thinking*). The fact that the PPV dropped during the effectiveness assay does not invalidate efficacy data, therefore it is not misleading to say that we ended up with a “very fast and cost-effective screening method”.

L.155-160 this passage is redundant since it reiterates information given in the Introduction.

The sentence was replaced by this: “Fig 1 shows the training program and experimental design.”.

L.160-164 (up to „…from each other…”) this passage should be included into Material section (collecting odor samples).

A new sub section called “Specimen collection for in vitro work” was created in Methods, between Sample Size and Dog Training. The paragraph in lines 160-164 was moved there.

L.164-178 this passage should be shifted to line 211 (Dog training).

Done.

L.166. Fig.1. showing the training phases should be cited here.

Done.

L.196-200 Should be moved to the Material section - collecting odor samples.

Done.

L.202-208 A separate paragraph on Ethical permission, should be moved to the beginning of the M&M section. The passage in lines 202-208 has nothing to do with Sample size.

Done.

L.235 My suggestion: „ ……could not bite, lick or touch ….”.

The new line reads this way: “…and the difference between both was that animals allocated to experimental groups could not bite, lick, or touch D1 or D2,…”.

L.265-270 Statistical analyses should be the last separate paragraph of the M&M section.

Done.

L.447-449 Rewarding some but not all correct alerts during the effectiveness assay cannot be considered as human error. In real screening scenario neither the experimenter nor the handler does know if the dog's response was correct or wrong (true double blind procedure). Therefore during real screening scenario, basically no rewarding have to be applied. Increasing the false negative alerts, or false positive alerts as a consequence of not rewarding the dog for EVERY correct alert on real people, is one of the critical points of the canine screening. It remains an open question, if the dogs that are subjected to a sustaining/improving training in order to reduce false negative and false positive alerts, using odor samples and lineup, would equally well alert on live people (in long term). There is something like context dependent olfactory learning that has to be taken into consideration.

The expression “human error” was taken off the sentence, which now reads this way: “. One explanation was handlers rewarding some but not all alerts during the effectiveness assay but, in fact, rRT-PCR results showed that many correct alerts passed unrewarded; it confused the dogs and caused even more false negative alerts.”.

L.462 „bred” instead of „created”.

The new line reads this way: “Second, all six dogs were successful as medical detectors despite belonging to breeds not intended specifically for scent-detection.”:

L.463 „suitability” instead of „excellence”.

See answer to L.465 below.

L.464 add „concentration ability”.

See answer to L.465 below.

L.465 „individual dogs” instead of „individual prospect”.

The new line reads this way: “It supports recent data showing that, more than the breed, the best predictors of suitability for medical detection dogs are the levels of motivation, stamina, determination, resilience, and concentration ability of the individual dog [42,43].”:

L.468 „…the canine recognizes variations….” (?) – rather ignores variations and indicates regardless of distracting odors.

The new line reads this way: “This process, called generalization, applies to learning theory, and in reference to scent-detection means that the canine ignores variations of the positive stimulus and indicates its source regardless of distracting odors [44].”.

L.469 „source” is redundant here. I suggest: „indicates the target odor regardless....”.

Actually, we trained our dogs to alert on the SOURCE of the target scent instead of the odor itself. It is useful for outdoors scent-work, where a moving odor causes the dog could alert before arriving to the source. On the scent-line with humans, it could cause the dog to alert on the wrong individual when wind is blowing towards the dog.

L.470 but is surprising in view of other medical detection dog training e.g. for cancer detection.

The word “surprising” was eliminated. The new sentence reads this way: “Generalization after exposure to just three specimens is to be expected if errorless learning principles are the foundations of training [31].”.

L.475-478 This sentence should be rewritten since it is confusing. It is not a problem of using urine as both positive (cancer) and negative (healthy stimulus), but the problem of using the same samples (donors) for the training and testing. Also, Ellier et al. 2014 was not the only study that recommended using odor samples from many donors and conducting the training and testing using different samples (donors).

The new sentence reads this way: “However, experimental evidence in favor of such hypothesis is scarce, and most citations refer to a work in which urine was employed as positive and negative stimulus during training [48].”.

L.480-481 „ …..using the same type of secretion during the foundational training does not favor errorless discrimination learning…..” (1) - what is the foundational training? - perhaps initial training ?, (2) – finally the dogs have to discriminate odor samples from sick vs healthy humans and not sick humans vs sterile saline solution. The dogs may be perfect at what the authors label as „errorless dicrimination training”, but may show poor performance in real screening scenario.

True, but in vivo experiments (efficacy trial, phase 3) demonstrated a very high effect size for every diagnostic metrics, including PPV. We did not invent errorless discrimination learning, it is actually the product of a very solid scientific work, as cited in the manuscript. The fact the dogs failed during the effectiveness assay does not affect the truth of errorless learning because the reward system was changed. To eliminate the term “foundational training” the new sentence reads this way: “Beyond the many variables specific to certain diseases and specimens that might be responsible for a greater level of difficulty for the dog, using the same type of secretion when the dogs are first trained for scent-detection does not favor errorless discrimination learning [31,32,45].”.

L.483 what stands for Effect size ? perhaps simply "The different diagnostic metrics"?

Just to answer the question of R1: effect size is a mathematical term that refers to the magnitude of a difference between two groups or, simply put, the most important result of most experiments. In this study, the effect size is the difference in diagnostic performance between the index test (K9 scent-detection) and the gold standard (rRT-PCR). To validate our dx test, we used these metrics, or effect sizes: SEN, SPC, PPV, NPV, ACC, and LR. Reviewer 1 is pointing correctly that we have created confusion by calling the values of these metrics “effect sizes”, because one thing is the name of a parameter or a metric or an effect size, and other is its numerical value or magnitude. In the new version, all references to the numeric result of any metric (aka effect size) are called “value”, “magnitude”, or “mean”.

L.488-490 „…Had they been scenting in search of odors other than their target…” please rewrite to be more clear. Perhaps: „..Had they been alerting to odors other than the target odor…”?

The new sentence reads this way: “Had they been alerting to odors other than their target odor, *SPC* would have been very low, and it was also >95%.”.

L.490-492 – please rewrite to be more clear.

The new sentence reads this way: “While *SEN* and *SPC* refer to the index test (canine scent-detection), predictive values quantify the probability that the participants truly had or not COVID-19, taking the reference standard (rRT-PCR) as the truth.”.

L.500-508 I suggest to delete the passage in lines 500-508 because canine learning as such goes beyond the scope of this study.

Done. The reader is directed to references 49-53 in lines 497-499: “Previous knowledge of dog behavior and cognition also suggest that our dogs were not relying in memory when they detected SARS-CoV-2 and discriminated it among so many other odoriferous cues [44,49-54].”.

L.560 while infectious diseases could be detected in seconds, could they also be controlled in seconds?

It is if, as in South Korea and New Zealand, positive patients are isolated immediately. The chain of transmission stops right there. That said, we understand that R1 is pointing to the fact that the data had nothing to do with controlling the transmission of COVID-19. Therefore, the words “and control” were eliminated. The new sentence reads this way: “With improved training methods, canines could, in the near future, provide a sensitive and effective method to detect infectious diseases in a matter of seconds.”.

Fig 1. „…..training phases and experimental design were planned…”? – or were conducted ?.

The new title of Fig 1 reads this way: **Fig 1. Efficacy studies. Flow chart depicting the order in which training phases and experimental design were conducted.**

Table 3 The term Effect size may be confusing. I suggest to delete (%) in the first left side column and insert (%) instead of Effect Size in column captions.

Done. The title of all columns was modified to reflect the difference between the effect size (metric) and its magnitude (numerical value).

In my opinion this is an interesting study that is worth publishing, but would definitely benefit from a better preparation of the second revision, including restructuring and extensive editing of English. Some parts of the Discussion are still not clear or difficult to follow. Therefore I recommend minor revision before final acceptance.

Done. After introducing the changes described above, the manuscript was reviewed and edited (in style) by a US native scientist who is familiar with the methods employed in this study.
